# Depression among Endometrial Cancer hospitalizations - Preliminary results of a nationwide retrospective study

**DOI:** 10.1192/j.eurpsy.2022.560

**Published:** 2022-09-01

**Authors:** P. Vieito, A.R. Ferreira, M. Gonçalves-Pinho, F. Costa, M. Coelho, A. Freitas, L. Fernandes

**Affiliations:** 1 University of Porto, Faculty Of Medicine, Porto, Portugal; 2 CINTESIS - Center for Health Technology and Services, Faculty of Medicine, University of Porto, Department Of Community Medicine, Information And Health Decisions Sciences, Porto, Portugal; 3 Faculty of Medicine, University of Porto, Department Of Clinical Neurosciences And Mental Health, Porto, Portugal; 4 Centro Hospitalar do Tâmega e Sousa, Departamento De Psiquiatria E Saúde Mental, Penafiel, Portugal; 5 Faculty of Medicine, University of Porto, Department Of Community Medicine, Information And Health Decision Sciences (medcids), Porto, Portugal; 6 Centro Hospitalar do Tâmega e Sousa, Departamento De Ginecologia E Obstetrícia, Penafiel, Portugal; 7 Psychiatry Service, Centro Hospitalar Universitário De São João, Porto, Portugal

**Keywords:** Endometrial Cancer, surgery, Depression, Administrative data

## Abstract

**Introduction:**

Uterine cancer is the most common gynecologic malignant neoplasm in developed countries. While depression is up to 3-5 times more common in patients with cancer than in the general population, literature is still limited regarding the relation between Endometrial Cancer and depression.

**Objectives:**

To analyze Depression among Endometrial Cancer hospitalizations in mainland Portuguese public hospitals (2008-2015).

**Methods:**

A retrospective observational study was conducted using administrative data from all hospitalizations in Portuguese mainland public hospitals between 2008-2015. All women’s hospitalizations(≥18 years) with a primary diagnosis of Endometrial Cancer (ICD-9-CM 182.x) were selected. Secondary diagnosis of depression was identified with ICD-9-CM 296.2x, 296.3x and 311x codes. Surgical procedures codes 68.4x, 65.6x, 40.3x, 40.5x, 68.6x, 68.9x and 68.8x were used to divide the hospitalizations into surgical vs non-surgical. Groups were compared with Pearson Chi-square test and crude odds ratio(OR) was used to estimate the association between surgery and depression.

**Results:**

From 10227 hospitalizations with a primary diagnosis of Endometrial Cancer, 533 had a registry of depression(5.2%). Annual depression frequency rose from 2.0% (2008) to 8.3% (2015). Among patients with a record of depression, 73.2% had surgery. Women who had surgery were significantly more likely to have registered depression (p<0.001). The OR for depression in surgical vs non-surgical patients was 1.73 (95%IC:1.42-2.10).

**Conclusions:**

Patients hospitalized due to Endometrial Cancer and submitted to surgery had almost two-fold more risk of having a registry of depression. This trend reinforces the importance of early depression screening of these patients, enabling the implementation of timely therapeutic strategies before and after surgery.

**Disclosure:**

No significant relationships.

